# Pounding score of intracranial lipomas

**DOI:** 10.1002/ccr3.7948

**Published:** 2023-09-22

**Authors:** Yudai Aikawa, Takanobu Sato, Ryo Ichibayashi

**Affiliations:** ^1^ Division of Emergency Medicine, Department of Internal Medicine Toho University Medical Center, Sakura Hospital Chiba Japan

**Keywords:** diagnostic imaging, headache, intracerebroventricular, lipomas

## Abstract

Most intracranial lipomas are asymptomatic, but headache is the most common symptom. The pounding score is sometimes high. Therefore, it is necessary to monitor imaging findings in parallel with the treatment of migraine.

## Graphical abstract

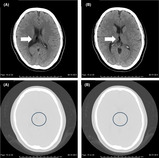

A 29‐year‐old woman presented to the emergency department with a 2‐month‐old headache. Her headache was a unilateral throbbing headache like she had never experienced before. Her headache lasted more than 4 h and was accompanied by vomiting. Otherwise, she had no abnormal neurological findings. She has a history of obsessive‐compulsive disorder. She is on regular oral paroxetine hydrochloride and cloxazolam. She gained 30 kg weight in 4 years. So far, she has not seen a doctor because of her headache, although she has acknowledged it many times. She had blood pressure of 120/80 mmHg, pulse of 60/beat, body temperature of 35.0°C, pupil of 5 mm/5 mm, and bilateral light reflex. Her Glasgow Coma Scale was 15 with no neck stiffness. Her blood tests showed no abnormalities. She underwent a head CT to rule out subarachnoid hemorrhage. Her head CT showed no intracranial hemorrhagic lesions. However, she had low‐density structures in the right lateral ventricle and third ventricles. CT values ranged from −20 to −80. When the head CT was set to the condition of the lung field, it was visually confirmed that it was not air (Figure 1A). As a result, she was diagnosed with an intraventricular lipoma. The pounding score was 5 points, suggesting the possibility of migraine.^1^ After her symptoms improved with analgesics (She received 1000 mg of Acetaminophen through an intravenous injection.), she was sent home for outpatient follow‐up. Intracranial lipoma is a rare benign tumor. Most are located in the midline within the cranium. Lipomas around the corpus callosum are associated with the hypoplasia and agenesis of the corpus callosum. Many cases are asymptomatic, with headache being the most common symptom. It is often found incidentally on head CT. Intracranial lipomas with headaches are found not only in the ventricle but also in other sites.^2^ At first glance, it can be mistaken for air mixed in the skull. When the CT value is measured, it shows a numerical value from 0 to −100 and is diagnosed as adipose tissue. It is rarely removed by surgery and is observed. The pounding score is a scale for diagnosing migraine. There are no reports of its use for intracranial lipoma. In our case, lipomas were found in the proper and third ventricles, and the right lateral ventricle was more significant than the left (Figure 1B). Headache due to intracranial lipoma was also considered. Still, from the result of the pounding score, it was diagnosed as a headache due to a migraine. Intracranial lipomas that have complained of headaches may include cases of migraine. Therefore, even if the intracranial lipoma is diagnosed, measuring the pounding score and differentiating migraine is necessary. On the contrary, the causal relationship between intracranial lipoma and headache has not been elucidated. For this reason, even if symptoms improve with analgesics, follow‐up imaging is necessary when ventricular laterality, giant lipoma, and unexplained headache persist.

**FIGURE 1:**
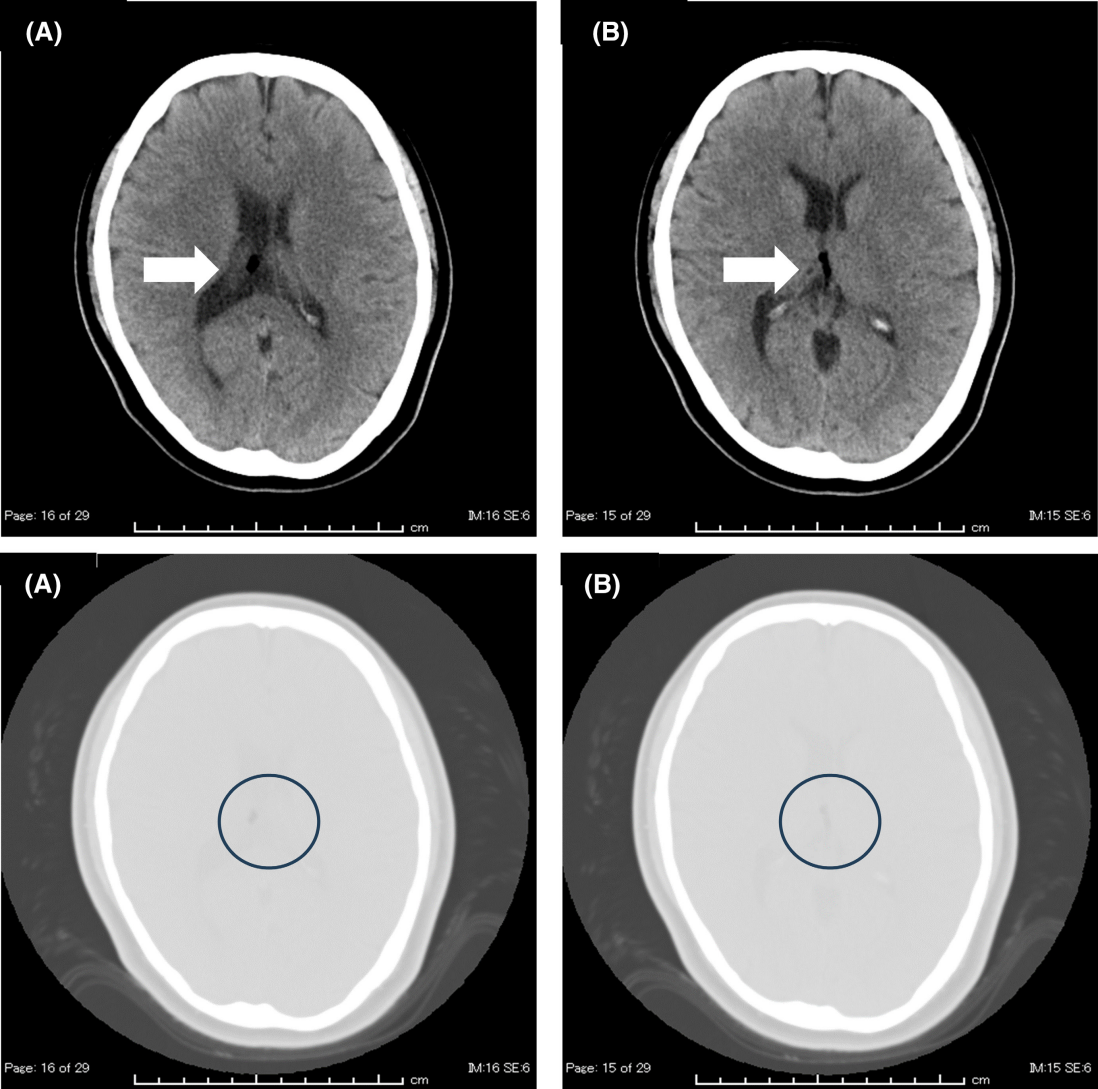
(A, B) Head CT. Upper row: (A) is a lipoma in the right lateral ventricle and (B) in the third ventricle. (white arrow). Lower row: It can be confirmed that both A and B are not air under lung field conditions. (black circle).

## AUTHOR CONTRIBUTIONS

**Yudai Aikawa:** Writing – original draft; writing – review and editing. **Takanobu Sato:** Writing – review and editing. **Ryo Ichibayashi:** Supervision; writing – review and editing.

## FUNDING INFORMATION

The author(s) received no financial support for this article's research, authorship, and/or publication.

## CONFLICT OF INTEREST STATEMENT

The authors have no conflict of interest to disclose.

## CONSENT

Written informed consent was obtained from the patient to publish this report by the journal's patient consent policy.

## Data Availability

The data presented in this study are available on request from the corresponding author. The data are not publicly available due to privacy and ethical considerations.
